# The effects of acupuncture treatment on the right frontoparietal network in migraine without aura patients

**DOI:** 10.1186/s10194-015-0518-4

**Published:** 2015-04-18

**Authors:** Kuangshi Li, Yong Zhang, Yanzhe Ning, Hua Zhang, Hongwei Liu, Caihong Fu, Yi Ren, Yihuai Zou

**Affiliations:** Department of Neurology and Stroke Center, Dongzhimen Hospital, The First Affiliated Hospital of Beijing University of Chinese Medicine, Beijing, 100700 China

**Keywords:** Acupuncture, Functional magnetic resonance imaging (fMRI), Migraine without aura (MWoA), Right frontoparietal network (RFPN), Resting-state

## Abstract

**Background:**

Functional and structural abnormalities in resting-state brain networks in migraine patients have been confirmed by previous functional magnetic resonance imaging (fMRI) studies. However, few studies focusing on the neural responses of therapeutic treatment on migraine have been conducted. In this study, we tried to examined the treatment-related effects of standard acupuncture treatment on the right frontoparietal network (RFPN) in migraine patients.

**Methods:**

A total of 12 migraine without aura (MWoA) patients were recruited to undergo resting-state fMRI scanning and were rescanned after 4 weeks standard acupuncture treatment. Another 12 matched healthy control (HC) subjects underwent once scanning for comparison. We analyzed the functional connectivity of the RFPN between MWoA patients and HC subjects before treatment and that of the MWoA patients before and after treatment. Diffusion tensor images (DTI) data analyzing was also performed to detect fiber-related treatment responses.

**Results:**

We observed significantly decreased FC in the RFPN and that the decreased FC could be reversed by acupuncture treatment. The changes of FC in MWoA patients was negatively correlated with the decrease of visual analogue scale (VAS) scores after treatment. This study indicated that acupuncture treatment for MWoA patients was associated with normalizing effects on the intrinsic decreased FC of the RFPN.

**Conclusions:**

Our study provided new insights into the treatment-related neural responses in MWoA patients and suggested potential functional pathways for the evaluation of treatment in MWoA patients. Future studies are still in need to confirm the current results and to elucidate the complex neural mechanisms of acupuncture treatment.

## Background

As the most prevalent neurological disorder, migraine is affecting more than 100 million people in Europe [1] and the USA [2]. Migraine ranks in the top 20 of the most disabling medical illnesses globally, and has substantial effects on the quality of life of patients and their families and on health costs [3]. It has attracted more and more attention worldwide as a public health issue because of its high prevalence, frequent attack history, significant medical burden, and a serious reduction in quality of life [4].

Migraine is typically associated with pain and its regulation. A series of multiple functional and structural abnormalities within pain related resting-state brain networks in migraine patients have been confirmed by previous functional magnetic resonance imaging (fMRI) studies [5-9]. Altered functional connectivity mainly distribute in the right frontoparietal network (RFPN), the default mode network (DMN), the sensorimotor network (SMN), the silence network, the periaqueductal gray networks, and so on. As a powerful tool to map intrinsic brain activities, fMRI also provides means to elucidate the possible neural mechanisms associated with successful treatment for certain diseases. Previously, the neural responses of medical therapies on major depression [10], stroke [11] and some other diseases have been confirmed by fMRI studies. However, few studies focusing on the neural responses of therapeutic treatment on migraine have been conducted.

A large body of clinical researches [12-14] and systematic reviews [15] have confirmed the successful effects of acupuncture for migraine. Existing results suggest that acupuncture is able to alleviate headache degree and/or improve the quality of life and it is as effective, if not more effective, as prophylactic drug treatment. Converging evidence from recent fMRI studies have demonstrated that immediate acupuncture stimulation evoke certain neural activities of different brain regions and networks in both healthy subjects and patients [16,17]. Thus, we believe that acupuncture could be applied as a therapeutic treatment in fMRI studies investigating the neural responses associated with treatment in migraine patients.

To our knowledge, only one study reported the neural responses of acupuncture treatment in migraine patients [18]. Their results indicated that acupuncture treatment evoked cerebral response in the pain matrix, the lateral pain system, the medial pain system, the DMN, and some cognitive components of the pain processing system. However, changes of functional connectivity between pain related brain regions and networks were not well explored in this study. Moreover, the acupuncture treatment involved only four acupoints which could not be regarded as comprehensive acupuncture treatment for migraine patients.

Given the fact that multiple functional and structural abnormalities exist in migraine patients and that acupuncture is an effective treatment for migraine, we hypothesized that acupuncture treatment would modulate the altered brain function of specific brain regions and networks. This hypothesis is of particular interest in respect of elucidating the mechanisms of treatment related neural responses in migraine patients. To test our hypothesis, we examined the effects of 4 weeks standard acupuncture treatment on acupuncture-naive migraine without aura patients. We focused on changes of functional connectivity in the RFPN, an important and dominant brain network strongly related to the processing and regulating of pain [19]. Relationships between functional changes and migraine symptom improvements were also examined. We also performed diffusion tensor images (DTI) data analyzing to detect fiber-related treatment responses.

## Methods

### Ethics statement

Written informed consents were obtained from all subjects. The data was analyzed anonymously. All research procedures were approved by the ethical committee of Dongzhimen Hospital affiliated to Beijing University of Chinese medicine and conducted in accordance with the Declaration of Helsinki.

### Subjects

A total of 12 MWoA patients (10 females, mean age: 28.1 ± 6.8 years) were recruited from Dongzhimen Hospital. All 12 patients met the following inclusion criteria: diagnosed of MWoA according to the classification criteria of the International Headache Society; between 18 and 60 years old; right-handed; 2 to 6 migraine attacks per month during the last 3 months; with a history of migraine longer than 1 year; without history of prophylactic or therapeutic medicine during the last 3 months; without history of acupuncture treatment; without history of smoking, alcohol or drug abuse; without history of long-term analgesics consumption; without history of dysmenorrhea or other chronic painful disease; with education background of more than 10 years; without any MRI contraindications; the informed consent form signed. Patients were excluded if they met any of the criteria below: other types of migraine; the first occurrence of migraine attack appeared after 50 years old; concurrence of neurological disease or psychiatric disorder; with diseases of the cardiovascular system, liver, kidney, or hematopoietic system; pregnant or lactating women; participation in other clinical trials.

Another 12 right-handed normal subjects matched in age, gender and education level were recruited to serve as healthy control (HC). All 12 HC subjects passed normal neurological examination and had no history of migraine or other neurological disease, psychiatric disorder or any MRI contraindications. The demographic and clinical information of MWoA patients and HC subjects was summarized in Table 1.

**Table 1:** **The demographic and clinical information of MWoA patients and HC subjects**

**Items**	**Healthy control (N=12)**	**MWoA patients before treatment (N=12)**	**MWoA patients after treatment (N=12)**
Gender (male/female)	2/10	2/10	2/10
Age (years)	29.8 ± 7.2	28.1 ± 6.8^#^	28.1 ± 6.8
migraine history (months)	/	47.3 ± 42.1	47.3 ± 42.1
Educational level (years)	14.8 ± 5.5	15.2 ± 4.4^#^	15.2 ± 4.4
VAS scores	/	5.5 ± 1.3	2.7 ± 0.7^*^
Frequency of migraine attacks (times/month)	/	4.5 ± 1.1	1.9 ± 0.7^*^
Duration of migraine attacks (days/month)	/	6.1 ± 3.2	4.3 ± 1.8^*^

Note: ^#^results from independent sample t-test of the comparison between MWoA patients and HC subjects, P > 0.05.

*results from paired t-test of the comparison in MWoA patients before and after treatment, P < 0.05.

### Acupuncture treatment

All MWoA patients received standard acupuncture treatment for 4 weeks. The acupuncture treatment course was established according to Chinese guidelines of acupuncture for migraine patients. The following acupoints were selected for needling: bilateral Sizhukong (SJ23), Shuaigu (GB8), Fengchi (GB20), Taiyang (EX-HN5), Hegu (LI4), Taichong (LR3), Waiguan (SJ5), Yanglingquan (GB34), and Zulinqi (GB41). All acupoints were located according to the WHO standard acupuncture point locations in the Western Pacific Region. The disposable stainless steel acupuncture needles (0.25 × 40 mm, Ande Co., Guizhou, China) were inserted in an appropriate angle to a depth of 1.5-2.5 cm. Each acupuncture needle was twisted until the patient felt a de-qi sensation and retained for 30 min. Acupuncture treatment was performed by an independent practitioner with 7 years of clinical experience. The acupuncture treatment took place 5 times per week (form Monday to Friday) and lasted for 30 min every time.

To ensure the pure effect of acupuncture treatment, all MWoA patients were told not to take any medication or preventive treatment during the whole study. In case of intolerable migraine attacks, patients could take analgesics they commonly used without the effect of migraine prevention and should record the doses and effects.

### fMRI data acquisition

All MWoA patients received two separate resting-state fMRI scanning, before and after the acupuncture treatment course respectively. The HC subjects participated in one resting-state scanning as control.

FMRI images were acquired using a 3.0 T MRI scanner (Siemens, Sonata, Germany). During scanning, subjects remained in the supine position with their heads immobilized by a custom-built head holder to prevent head movements. Subjects wore earplugs throughout the experiment to attenuate MRI gradient noise. Thirty-two axial slices (field of view = 225 mm × 225 mm, matrix = 64 × 64, thickness = 3.5 mm) parallel to the anterior-posterior commissure plane and covering the whole brain were obtained using a T2-weighted single-shot, gradient-recalled echo planar imaging sequence (repetition time = 2000 ms, echo time = 30 ms, flip angle = 90°). Prior to the functional run, high-resolution structural information on each subject was also acquired using 3D MRI sequences with a voxel size of 1 mm3 for anatomical localization (repetition time = 1900 ms, echo time = 2.52 ms, flip angle = 90°, matrix = 256 × 256, field of view = 250 mm × 250 mm, slice thickness = 1 mm).

The DTI data were obtained with a single-shot, echo-planar imaging sequence. The diffusion sensitizing gradients were applied along 30 non-collinear directions (b = 1000 s/mm^2^) with an acquisition without diffusion weighting (b = 0 s/mm^2^). The imaging parameters were 80 contiguous axial slices (repetition time = 18000 ms, echo time = 94 ms, flip angle = 90°, matrix = 160 × 160, field of view = 256 mm × 256 mm, slice thickness = 1.5 mm).

In the current fMRI study, for the MWoA patients, we set a rule that the acupuncture treatment should begin within 3 days after the first fMRI scanning and the second scanning should be done within 3 days after the acupuncture treatment course ended. It has to be emphasized that if there was a migraine attack during the due date of scanning, the fMRI scan should be postponed for 72 hours. Another rule for female subjects in both the migraine and control groups was that all fMRI scanning should be done at least 3 day away from their menstrual period.

### FMRI data processing and analyzing

The fMRI data included resting scans of HC subjects and MWoA patients. The HC subjects were scanned once and the scans of MWoA patients were collected before and after the acupuncture treatment. The first 10 time points of all datasets were discarded to avoid unstable magnetization and to ensure the participants adapted the scanning circumstance. All data was preprocessed using the DPARSFA 2.3 software [20]. The images were firstly corrected for acquisition delay between slices by aligning to the first image of each session for motion correction using method of voxel specific head motion [21]. Afterwards, fMRI data was registered to the templates created by T1 images segmented by DARTEL method. Data was further processed with spatial normalization based on the MNI space and resampled at 2 mm × 2 mm × 2 mm. All subjects’ had less than 1.5 mm head motions and less than 1.5° rotation in any direction. In addition, a Gaussian kernel with a full width at half-maximum of 6 mm was used to smooth the images in order to reduce noise and residual differences. Finally, we detrended the data to minimize the influence of magnetic machine temperature rise.

Data after preprocessing was analyzed by independent component analysis using the GIFT software. Then, the best goodness-of-fit score to the frontoparietal network template [19,22] was calculated by the way of goodness-of-fit [23] after removing the components not related to the spectrum of resting-state networks (spectrum of resting state networks was 0.01-0.08Hz). The images of component with best goodness-of-fit score were normalized to Z-scores with Fisher’s r-to-z transformation to acquire the entire brain Z-score map of each subject.

For group-level analyses, the functional connectivity was conducted by two-sample T-test and paired T-test using SPM8 software (two-tailed, p < 0.05, Monte Carlo Simulations correction). The reported statistics were color-coded and mapped in Talairach space. Finally, for the regions of interest in which MWoA patients showed decreased functional connectivity before acupuncture treatment in contrast with HC and significant increased functional connectivity after acupuncture, the Z values of each patient in these regions were extracted, averaged and regressed against patients’ visual analogue scale (VAS) scores.

### DTI data processing and analyzing

The data processing and analyzing were mainly carried out using FMRIB Software Library (FSL) and Analysis of Functional NeuroImage (AFNI) software. Firstly, the original DTI data was deobliqued to ensure the images were accord with anterior-posterior commissure line. Then, the Brain Extraction Tool in FSL software was used for brain extraction. And the eddy current distortion and head motion of raw diffusion data were corrected using FSL software. After that, b-vectors was rotated to accord with the results of eddy-correct [24]. Finally, tensor was computed using AFNI software.

We used the DTITK and DTI VISTA software in this procession. The details of DTITK processing steps were as follows [25,26]: firstly, initial template was bootstrapped from each subject’s tensor and IIT3 mean template image to achieve the optimal spatial normalization. Secondly, tensors were affinely registered to the initial template using a similarity metric known as Euclidean Distance Squared. Thirdly, each subject’s tensor after affine registration was deform ably aligned to the initial template to improve alignment quality by removing size or shape differences in the local structures. Then, a matrix combining the affine transformation and deformable transformation was applied on the original tensors. At last, tensors after the above processing were combined to produce a final average template with characteristics of all subjects. After producing the average template, mean DTI data was transformed from average template by using the AFNI software. After that, DTI VISTA processing was applied.

We defined the following brain regions as regions of interest (ROI): brain regions that showed decreased functional connectivity in MWoA patients compared with HC before treatment (ROI1); brain regions that showed increased functional connectivity in MWoA patients after treatment compared with that before (ROI2); and the left precentral gyrus (ROI3). Fibers from the left thalamus to the above mentioned three ROIs were calculated by ConTrack algorithm [27]. The fibers that not corresponding with anatomical position were cut by the Quench software. Finally, we applied Brain Voyager QX to present the results.

## Results

### Treatment response

A total of 12 MWoA patients were involved in the acupuncture treatment and finished the treating course as planned. All patients reported the sensation of de-qi and no adverse invents happened. Compared with baseline assessments, the results of VAS scores, duration and frequency of migraine attacks showed significant decrease (P < 0.05) after 4 weeks acupuncture treatment (see Table 1).

### Resting-state results

**Figure 1:**
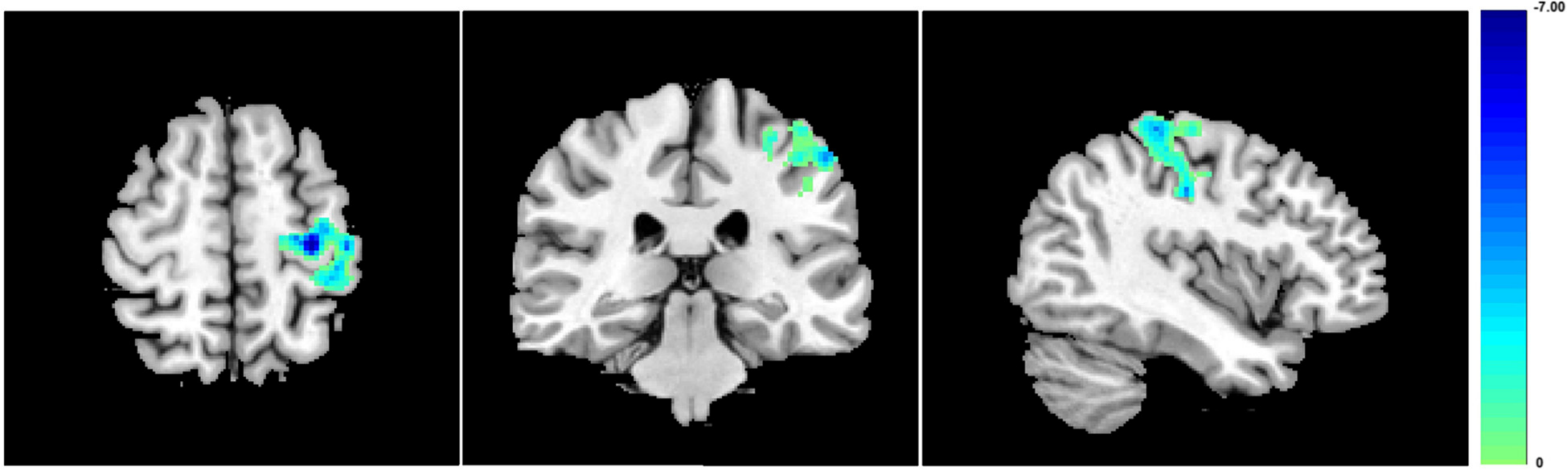
Compared with HC subjects, MWoA patients showed decreased functional connectivity in the left precentral gyrus, the left supramarginal gyrus, the left inferior parietal lobule, and the left postcentral gyrus. Results from two-tailed, p < 0.05, corrected by Monte Carlo Simulations, iterated 1000 times, and cluster size > 349 voxels.

**Figure 2:**
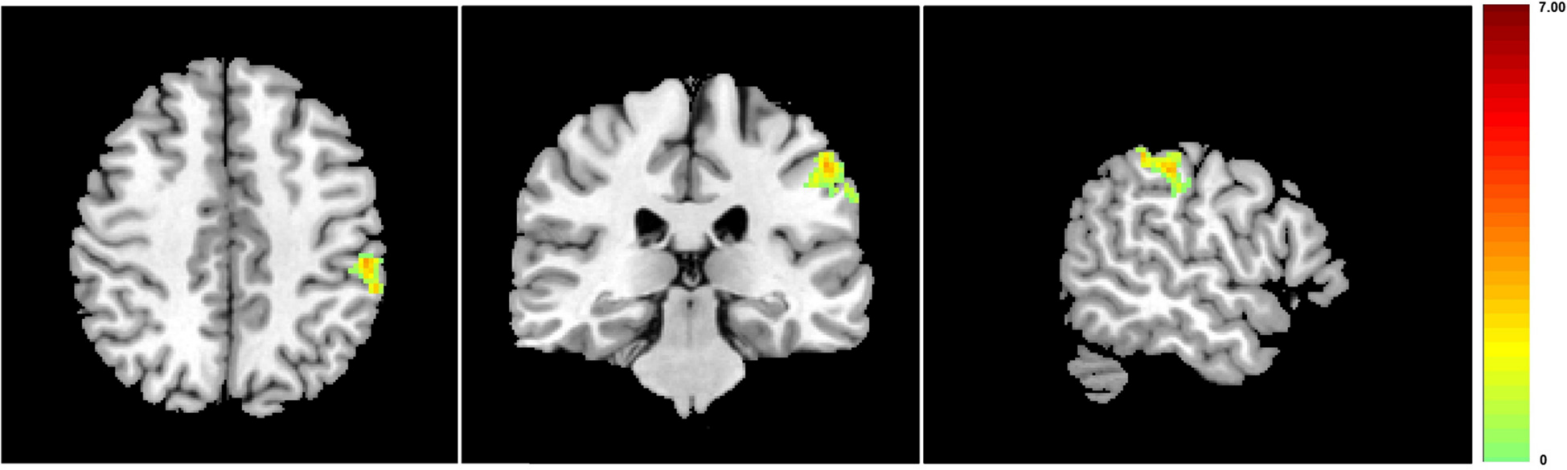
Compared with before acupuncture treatment, MWoA patients showed increased functional connectivity in the left precentral gyrus, the left inferior parietal lobule, and the left postcentral gyrus, after acupuncture treatment. Results from two-tailed, p < 0.05, corrected by Monte Carlo Simulations, iterated 1000 times, and cluster size > 349 voxels.

To investigate differences of functional connectivity with the RFPN between MWoA patients and HC subjects, we compared the data of HC subjects and MWoA patients before acupuncture treatment. MWoA patients revealed significantly decreased functional connectivity with the RFPN in the left precentral gyrus, the left supramarginal gyrus, the left inferior parietal lobule, and the left postcentral gyrus (Figure 1). And the decreased functional connectivity of brain regions in MWoA patients was negatively correlated with their VAS scores before treatment (Figure 2, P = 0.0494, R = -0.6289). No brain regions with increased functional connectivity in the MWoA patients were observed before treatment.

**Figure 3:**
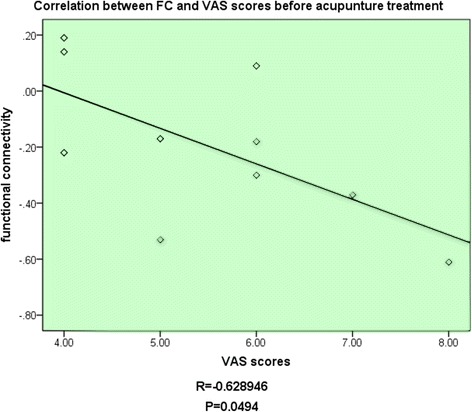
The decreased functional connectivity of brain regions in MWoA patients was negatively correlated with their VAS scores before treatment.

**Figure 4:**
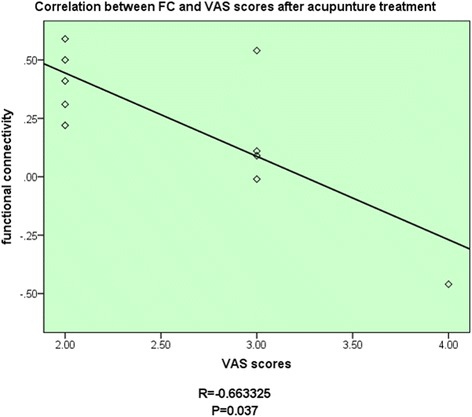
The increased functional connectivity of brain regions in MWoA patients was negatively correlated with the decrease of VAS scores after treatment.

For the MWoA patients, a comparison between before and after the acupuncture treatment was done to detect the changes of functional connectivity with the RPFN induced by acupuncture treatment. This was to evaluate the neural responses of acupuncture treatment in MWoA patients. After the acupuncture treatment, compared with that before, MWoA patients showed significantly increased functional connectivity with the RFPN in the left precentral gyrus, the left inferior parietal lobule, and the left postcentral gyrus (Figure 3). And the increased functional connectivity of brain regions in MWoA patients was negatively correlated with the decrease of VAS scores after treatment (Figure 4, P = 0.0370, R = -0.6633). No brain regions showed decreased functional connectivity with the RFPN when comparing the results after treatment with that before.

### DTI results

**Figure 5:**
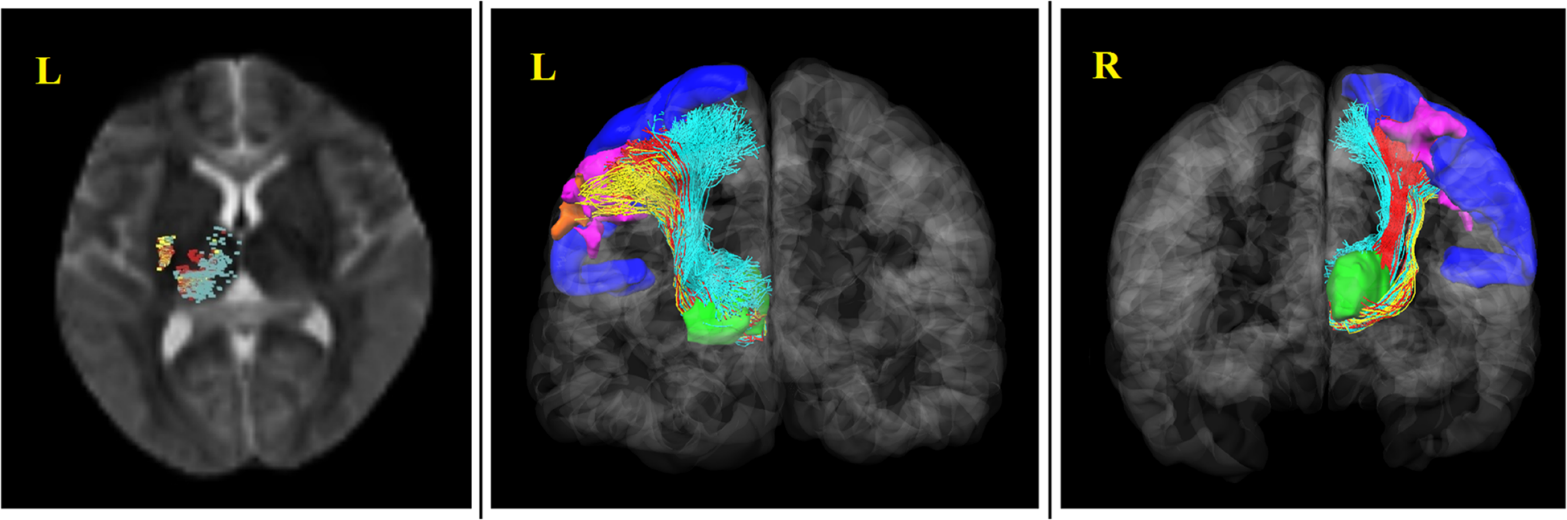
DTI tractography between the beginning region of interest to ROI1, ROI 2 and RIO3.The thalamus was selected as the beginning region of interest. ROI1 (pink): brain regions that showed decreased functional connectivity in MWoA patients compared with HC before treatment. ROI2 (orange): brain regions that showed increased FC in MWoA patients after treatment compared with that before. ROI3 (blue): the left precentral gyrus. The red, yellow and cyan fibers indicated fibers from the left thalamus to ROI1, ROI2 and RIO3 respectively.

Three probabilistic fibers were shown in Figure 5. The red, yellow and cyan fibers indicated fibers from the left thalamus to ROI1, ROI2 and RIO3 respectively. These three kind of fibers basically coincided with anatomical location. Moreover, these fibers passed the external capsule and posterior limb of the internal capsule which are in accord with the pathways of thalamic radiation. Most fibers passing through the posterior limb of the internal capsule terminated at the dorsal thalamus and fibers passing through the external capsule ended at the thalamus ventralis.

## Discussion

This is the first study to investigate the neural effects of standard acupuncture treatment on the functional connectivity of the RFPN in MWoA patients. We found decreased functional connectivity in the left precentral gyrus, the left supramarginal gyrus, the left inferior parietal lobule, and the left postcentral gyrus and that the decreased functional connectivity could be reversed by acupuncture treatment. Our results indicated that 4 weeks standard acupuncture treatment had normalizing effects on abnormally decreased functional connectivity in the RFPN in MWoA patients. We propose that the reversal of functional connectivity in the RFPN may lead to further interpretation of treatment-related neural responses in MWoA patients.

The RFPN is recognized as an important brain network that corresponds to perception, somesthesis, and pain [19]. The frontoparietal region played a dominant role in the formation and transmission of sensation. This region connected the primary sensory area with the secondary sensory area [28]. Other studies demonstrated that the frontoparietal lobe is closely related to cognitive processing, memory working and attention keeping [29,30]. Accordingly, chronic continually pain suffers in MWoA patients, invariably causing human attention, might change the functional connectivity of the RFPN. Some recent researches also indicated that the functional connectivity of the RFPN exist abnormality in patients suffering from chronic pain [31,32]. Our results revealed decreased functional connectivity between the RFPN and some other brain regions. These brain regions are involved in direct or indirect relation with pain. The postcentral gyrus, which is usually called the primary somatosensory cortex (SI), is included in the pain matrix and accepts pain signals straightly [33]. The precentral gyrus, which is called the primary sensorimotor cortex (MI), is typically associated with voluntary movement. Recent studies have found links between the SI and MI indicating that some sensory signals could transmit from the SI to MI [34]. Other studies also revealed abnormal nodal centrality in the MI in MWoA patients when compared with HC subjects [35,36]. The changes of functional connectivity in the supramarginal gyrus and the MI were also reported in nociception [7]. Taken together, the brain regions showed decreased functional connectivity with the RFPN in our study played important roles in pain processing and regulating. The decreased functional connectivity in these brain regions probably caused by hyperexcitability of pain pathway which led to disorders between the RFPN and its related external regions [37,38]. In addition, the average functional connectivity value of the brain regions which showed decreased functional connectivity negatively correlated with VAS scores of MWoA patients. This suggested that these pain related brain regions became more hypo-connected with the RFPN as the pain intensity increase. The decreased functional connectivity in MWoA patients revealed the modulations of brain functions associated with ongoing nociceptive stimulus. From the perspective of brain network, our current findings provided a conceptualization of MWoA as the dysfunctions of brain networks [7,8,39,40].

In the comparison study before and after acupuncture treatment, we found that acupuncture treatment had normalizing effects on abnormally decreased functional connectivity between the RFPN and its external eareas in MWoA patients. After a 4-week acupuncture treatment, MWoA patients showed increased functional connectivity in the left precentral gyrus, the left inferior parietal lobule, and the left postcentral gyrus with the RFPN. From the perspective of transformation, we assumed that the normalizing effects might be associated with the changes of relation among different brain networks. The RFPN and DMN are considered as two main components of human brain resting-state networks [19]. The RFPN is strongly involved in perception, somesthesis, and pain [19]. While the DMN is involved in self-referential processing, conscious, awareness, mind wandering, manipulation of episodic memories and semantic knowledge [41,42]. The right insula was considered as an important node of the RFPN in previous studies [43]. And the inferior parietal lobule relating to the processing of pain was regarded as an important part of the DMN [19,44]. The right insula and left inferior parietal lobule revealed strong functional connectivity in resting-state in a recent research [45]. Some study also stated that the higher functional connectivity of the RFPN within the region of the intraparietal sulcus would lead to greater intrinsic DMN connectivity to insula, which was outside of the classical boundaries of the DMN [46]. Fibers connecting the inferior parietal lobe and the insula have also been found [47]. In our study, the functional connectivity of inferior parietal lobule after acupuncture treatment significantly increased when compared with that before acupuncture treatment. While this area of MWoA patients showed decreased functional connectivity before acupuncture compared with HC subjects. Thus, we speculated that the DMN was connected with the RFPN through certain nodes and that the left inferior parietal lobule probably was included in these nodes. The theory that some treatment methods were able to change the functional connectivity of nodes in brain networks had been confirmed in recent researches [48,49]. Our study confirmed that the abnormally decreased functional connectivity of the RFPN could be reversed by acupuncture treatment. The functions of the precentral gyrus and the postcentral gyrs in the rehabilitation of migraine are still unclear. However, a recent study have found differences of precentral gyrus cortical thickness between migraine patients and HC subjects [50]. Besides, the precentral gyrus was also found as an abnormal nodal centrality which related to pain-processing [35]. Other researchers have revealed increased metabolism in migraine patients after acupuncture treatment by PET-CT [51]. Therefore, the therapeutic effects of acupuncture might be associate with the adjustment in these brain regions as well their related functions. In combination with the current knowledge of physiological functions of the RFPN, we propose that such reversal in the functional connectivity of the RFPN may lead to a beneficial impact on pain processing and regulating functions in MWoA patients. Moreover, the increased functional connectivity of brain regions in MWoA patients was negatively correlated with the decrease of VAS scores after treatment. The symptomatic improvement suggested that the changes of functional connectivity of the RFPN may act as objective indicators of the clinical responses of MWoA patients to different treatment. Accordingly, our study indicated that acupuncture could improve the functional connectivity of nodes between the RFPN and DMN to promote the rehabilitation of migraine. Besides, successful acupuncture treatment was associated with a normalizing effects on the functional connectivity of the left precentral gyrus, left inferior parietal lobule and left postcentral gyrus in MWoA patients.

A large number of studies have been conducted to investigate the structural changes in multiple DTI-derived indices in MWoA patients [52-55]. However, microstructural studies could not support the functional studies in anatomy. Therefore, we tracked the fibers’ path to support the results of fMRI in the current study. According to the anatomical position, thalamic radiation distributed in the broad cortex including the frontal lobe, the parietal lobe, the occipital lobe and the temporal lobe. At the same time, the thalamus was also an important structure in researches of acupuncture [56,57]. Therefore, the thalamus was selected as the beginning region of interest based on the anatomical structure and the effects of acupuncture. Then we defined brain regions that showed decreased functional connectivity in MWoA patients compared with HC before treatment as ROI1, brain regions that showed increased functional connectivity in MWoA patients after treatment compared with that before as ROI2, and the left precentral gyrus as ROI3. The results showed central thalamic radiation (showed in Figure 5, Cyan fibers), which started from the left thalamus to ROI3, corresponded to existing anatomy. Besides, fibers which started from the left thalamus to ROI2 overlapped with the central thalamic radiation. The fibers which started from the left thalamus to ROI1 also coincided with the central thalamic radiation. The fibers entirely passed though the posterior limb of the internal capsule and external capsule. Previous studies showed that the superior and posterior thalamic radiation, which directly related to sensory, joined the posterior limb of the internal capsule [58,59]. Moreover, fibers passed through external capsule were generally considered to be involved in movement and coordination of movement [60,61]. In our studies, the fibers mostly terminated in the lower part of the sensory region and motor region in cerebral cortex. According to anatomy, the distribution of sensory region and motor region in cerebral cortex were inverted human form. Dominant region of sensorimotor in head and face were mostly in the lower part of the motor cortex and sensory cortex. Therefore, we considered that the regions showed different functional connectivity, between MWoA patients and HC subjects, were related to the sensorimotor of head and face according to the anatomical location. Besides, the fibers, which started from the left thalamus to ROI3, also joined the thalamic radiation and ended at the MI and SI. Previous study showed that a conduction loop which could execute movement according to the sense existed between the motor cortex and sensory cortex [62]. To sum up, we speculate that the differences of functional connectivity between MWoA patients and HC subjects are related to the disorder of the sensorimotor pathways, and that changes of these pathways are possible one aspect of interpretations of treatment-related neural mechanisms. We also speculate that these pathways probably are the substance basis of functional connectivity in brain networks.

Our study has several limitations. First, we didn’t clearly confirm whether the changes of functional connectivity after treatment were due to the specific effects of acupuncture treatment, changes related to the natural course of the illness, a placebo effect, or some combination of these possibilities. Moreover, the DTI results only showed the contribution of fibers, but couldn’t explain the differences of fibers property between HC subjects and MWoA patients. Thus, comparing scores of fibers need to be applied on the follow-up studies. Finally, Further studies with larger sample size are still needed to confirm these results.

## Conclusions

In conclusion, the current study indicated that acupuncture treatment for MWoA patients was associated with normalizing effects on the intrinsic decreased functional connectivity of the RFPN. This study provided new insights into the treatment-related neural responses in MWoA patients and suggested potential functional pathways for the evaluation of treatment in MWoA patients. Future studies are still in need to confirm the current results and to elucidate the complex neural mechanisms of acupuncture treatment.

## Abbreviations

DMN: Default mode network
DTI: Diffusion tensor images
fMRI: Functional magnetic resonance
HC: Healthy control
MwoA: Migraine without aura
RFPN: Right frontoparietal network
ROI: Regions of interest
SMN: Sensorimotor network.
